# Blocking the PD-1/PD-L1 pathway in glioma: a potential new treatment strategy

**DOI:** 10.1186/s13045-017-0455-6

**Published:** 2017-04-07

**Authors:** Song Xue, Man Hu, Veena Iyer, Jinming Yu

**Affiliations:** 1 School of Medicine and Life Sciences, University of Jinan-Shandong Academy of Medical Sciences, 575 Mingfu Road, Jinan, 250200 Shandong China; 2 Department of Radiation Oncology and Shandong Province Key Laboratory of Radiation Oncology, Shandong Cancer Hospital Affiliated to Shandong University, 440 Jiyan Road, Jinan, 250117 Shandong China; 3 Shandong Academy of Medical Sciences, Jinan, China; 4 Hematology-Oncology, University of Toledo Medical Center, 1325 Conference Drive, Toledo, OH 43614 USA

**Keywords:** PD-1, PD-L1, Expression, Immunosuppressive, Treatment, Glioma

## Abstract

Gliomas are the most common type of primary brain tumor in adults. High-grade neoplasms are associated with poor prognoses, whereas low-grade neoplasms are associated with 5-year overall survival rates of approximately 85%. Despite considerable progress in treatment modalities, the outcomes remain dismal. As is the case with many other tumors, gliomas express or secrete several immunosuppressive molecules that regulate immune cell function. Programmed death-ligand 1 (PD-L1) is a coinhibitory ligand that is predominantly expressed by tumor cells. The binding of PD-L1 to its receptor PD-1 has been demonstrated to induce an immune escape mechanism and to play a critical role in tumor initiation and development. Encouraging results following the blockade of the PD-1/PD-L1 pathway have validated PD-L1 or PD-1 as a target for cancer immunotherapy. Studies have reported that the PD-1/PD-L1 pathway plays a key role in glioma progression and in the efficacy of immunotherapies. Thus, progress in research into PD-L1 will enable us to develop a more effective and individualized immunotherapeutic strategy for gliomas. In this paper, we review PD-L1 expression, PD-L1-mediated immunosuppressive mechanisms, and the clinical applications of PD-1/PD-L1 inhibitors in gliomas. Potential treatment strategies and the challenges that may occur during the clinical development of these agents for gliomas are also reviewed.

## Background

Gliomas account for 51.4% of all primary brain tumors and are thus the most common primary brain tumor in adults [1]. The World Health Organization (WHO) classifies gliomas as low-grade gliomas (LGGs) and high-grade gliomas (HGGs) according to aggressiveness. The 5-year overall survival (OS) rate of LGG patients is approximately 85%. However, the survival rate of HGG patients is less than 5% with the standard treatment of total surgical resection followed by radiotherapy and adjuvant chemotherapy [2]. The ability of gliomas to induce local and systemic immunosuppression limits the innate defense against tumor growth and the efficacy of adaptive immunotherapy and thus poses a significant challenge to the development of new therapies [3]. T lymphocytes have the potential to recognize antigens [4]. Immune checkpoints, particularly programmed cell death (PD)-1 receptor and its ligand (PD-L1), can suppress the activity of T lymphocytes [5]. The consequences of the binding of PD-1 to PD-L1 are apoptosis and the exhaustion of activated immune cells. Wei et al. [6] outlined the multitude of effects that are exerted on PD-1/PD-L1 by T cells, which induce the loss of proliferation and diminished cytokine production. In the past 5 years, immunotherapy with PD-1 and PD-L1 monoclonal antibodies (mAbs) has resulted in significant benefits, with durable responses and acceptable treatment-related toxicities in several types of tumors. Pembrolizumab and nivolumab (NIVO) (two checkpoint inhibitors that target PD-1) were approved by the Food and Drug Administration (FDA) for advanced melanoma therapy in late 2014 and for non-small cell lung cancer (NSCLC) therapy in March 2015 [7–10].

The success of immunotherapy in other cancers and the current understanding of the interaction between tumors and the immune system have generated increasing interest in the use of PD-1/PD-L1 inhibitors in the treatment of gliomas, particularly glioblastomas (GBMs). The first large phase III trial of NIVO in patients with GBM (CheckMate 143, NCT02017717) was initiated in January 2014 and is ongoing. Given that the PD-1/PD-L1 pathway is critical for downregulating the immune responses of gliomas, we reviewed its expression, the mediated immunosuppressive mechanisms, and the clinical applications of PD-1/PD-L1 inhibitors. We also considered potential treatment strategies and the challenges that may occur during the clinical development of these agents in gliomas.

## PD-L1 expression and prognosis value in gliomas

The expression of PD-L1 has been detected in glioma cell lines and tumor tissue. The expression of PD-L1 was detected in glioma cell lines as early as 2003 by Wintterle et al. [11]. They found that all 12 tested malignant glioma cell lines expressed PD-L1 mRNA. A subsequent study from Wilmotte et al. [12] revealed that the PD-L1 protein was also observed in 6/8 human astrocytoma cell lines. Immunohistochemical (IHC) studies have characterized PD-L1 expression in the cytoplasm and/or the cell membranes of glioma samples. The positive rate of PD-L1 protein expression was variable across different studies and ranged from 6.1 to 100%. Pooled analysis demonstrated an overall positive rate of PD-L1 protein expression of 44.72% (Table 1). A study with a small sample of 10 patients demonstrated that PD-L1 protein expression was detected in all 9 glioblastoma (WHO IV) specimens and in 1 mixed glioma (WHO III) specimen [11]. However, in a large series of human glioma samples involving 345 patients, the rate of PD-L1 expression positivity was found to be only 6.1%; specifically, positivity was found in 0/54 grade I/II, 0/47 grade III, and 21/244 grade IV gliomas (3 gliosarcoma and 18 GBM cases) [13]. However, there were substantial variations in the sample sizes, the ratios of the different pathological grades, the methods of preparing the tumor tissues, the antibodies used, and the diagnosis standards, including the expression patterns and positivity cut-offs, among these studies, which contributed to the biases in the results; these differences are characterized in Table 1. Therefore, further research to establish uniform standards is necessary.

**Table 1:** Summary of different assays for PD-L1 in the studies

Author (year)	Sample size	WHO grade	Assay	Material	Tissue samples	Antibody	Staining patterns	Cut-off^a^	Rate (%)^b^
Wintterle et al. (2003)	10	III + IV	IHC	Frozen sections	Full slides	5H1	NM	Presence of PD-L1 staining	100
Wilmotte et al. (2005)	54	II–IV	IHC	Frozen sections	Full slides	MIH1	Membranous/cytoplasm	Presence of PD-L1 staining	85.2
Yao et al. (2009)	48	I–IV	IHC	Frozen sections	Full slides	MIH1 (Ebioscience)	Membranous/cytoplasm	NM	NM
		I–IV	WB	Fresh tissues	Full slides	Anti-PD-L1 (R&D Systems)	NM	NM	75.0
Avril et al. (2010)	20	IV	IHC	PE	NM	Anti-PD-L1 (Clinisciences)	NM	>25%	45.0
Liu et al. (2013)	17	III + IV	IFC	Frozen sections	NM	Anti-human PD-L1 (558065; BD PharMingen)	NM	Presence of PD-L1 staining	76.5
Berghoff et al. (2014)	135	IV	IHC	PE	Full slides	5H1	Membranous	>5%	34.8
							Diffuse/fibrillary	Presence of PD-L1 staining	82.9
Nduom et al. (2015)	99	IV	IHC	PE	Tissue microarray	EPR1161(2) (Abcam)	Membranous	≥1%	60.6
Zeng et al. (2016)	229	I–IV	IHC	PE	Tissue microarrays	Rabbit anti-PD-L1	Membranous/cytoplasm	>5%	51.1
Garber et al. (2016)	345	I–IV	IHC	PE	Full slides	SP142 (Spring Biosciences)	Membrane	>5%	6.1
Pooled data	957								44.7

*Abbreviations*: *IHC* immunohistochemistry, *IFC* immunofluorescence histochemistry, *WB* western blot, *PE* paraffin-embedded specimens, *NM* not mentioned

^a^Cut-off value to determine positivity

^b^The rates of patients with glioblastomas with any PD-L1 protein expression on tumor cells

Recent studies have investigated the distribution of PD-L1 expression in glioma tissues. The patterns of PD-L1 expression were described as two main staining patterns: diffuse/fibrillary patterns and membranous patterns. Further analyses revealed no significant difference in the extent of diffuse/fibrillary or membranous PD-L1 expression between newly diagnosed and matched recurrent glioblastoma specimens [14]. Yao et al. [15] investigated the heterogeneity of PD-L1 expression in the subsites of glioma tumor tissues. The results revealed that PD-L1 expression was significantly greater at the edges of the tumors than in the tumor cores (*P =* 0.001), and this finding may be related to the invasion of gliomas. Upregulation of PD-L1 at the edge of the tumor forms a barrier between the tumor cells and cytolytic T cells; this phenomenon has been termed a “molecular shield” and contributes to the high rate of malignant infiltration and the escape from immune surveillance during invasion into the adjacent brain tissue.

To date, all of the relevant studies have demonstrated that the expression of PD-L1 in tumor tissues is correlated with glioma grade, which demonstrates that PD-L1 may be a candidate tissue biomarker for gliomas. Wilmotte et al. [12] found that PD-L1 staining in HGGs not only was more intense but revealed a greater proportion of positive cells (>30% stained cells) than in diffuse astrocytomas and oligodendrogliomas (18/33 vs. 1/12, *P* < 0.001, *χ* ^2^ test). In another study, in which 48 patients with gliomas were enrolled, western blot analyses revealed a significantly higher level of PD-L1 expression in HGGs (*n* = 24) than in LGGs (*n* = 24; *P* < 0.001) [15]. Baral et al. [16] reported that the expression of PD-L1 in freshly dissected human glioma tissues is correlated with the glioma grade. These findings suggest that the growth of the most malignant forms of glioma is promoted by the selection of tumor cells with a high level of PD-L1, which facilitates immune evasion. The selection of tumor cells with high levels of PD-L1 facilitates immune evasion and thus favors the growth of the most malignant forms of glioma. Thus, PD-L1 may be a potential biomarker and new therapeutic target for gliomas.

Major research efforts have been made to evaluate the prognostic value of PD-L1 in gliomas. In a study of 229 glioma (grades I–IV) patients, Zeng et al. [17] found no significant associations between PD-L1 expression and OS. Using the median survival time (12 months) as a cut-off point, these authors found that a high level of PD-L1 expression was significantly associated with the poor OS of patients who survived and were followed up over 12 months. Several studies have been conducted to determine the prognostic value of PD-L1 in the GBM subtype. However, the results have been inconsistent. Liu et al. [18] were the first to report that PD-L1 expression is a negative prognosticator for survival based on a very small series of 17 GBM cases. In a retrospective study by Berghoff et al. [14], who investigated PD-L1 expression in 563 GBMs and its correlation with patient outcome, the presence of diffuse/fibrillary PD-L1 expression was not associated with survival time in a cohort of 117 specimens of newly diagnosed GBM. Based on level 2 Agilent microarray gene expression, there was also no significant association between the PD-L1 gene expression level and OS in 446 patients with GBM from The Cancer Genome Atlas (TCGA) dataset. However, using level 3 Illumina RNASeq, Nduom et al. [19] found a significant association between PD-L1 gene expression and outcome in the same TCGA dataset. The median survival of the high-expression PD-L1 mRNA group was significantly shorter than that of the low-expression group (11.42 vs. 14.9 months, respectively; *P* = 0.023). The patients with high PD-L1 expression levels (dichotomized into low and high at the cut-off point 0.37) exhibited a significantly increased risk of death compared with patients with low expression levels (*P* = 0.0231), and PD-L1 was an independent factor that was negatively associated with survival (*P =* 0.0343). The prognostic influence of PD-L1 expression at the protein level was evaluated in a survival analysis of 94 GBM samples. Using the median as the cut-off point, the patients with >2.77% PD-L1-positive cells exhibited a trend toward worse OS (*P* = 0.066). However, when a cut-off of 5% positive cells was used, as has been used in numerous other studies of solid malignancies, high expression was associated with a significantly shorter survival (*P* = 0.0086), which confirmed the results obtained with the mRNA data. The different PD-L1 expression threshold levels and assay technologies (e.g., the Agilent microarray and the Illumina RNASeq) likely contributed to these inconsistent conclusions [20]. Therefore, further studies are required to determine the prognostic value of PD-L1 in gliomas.

## PD-L1-mediated immunosuppressive and upregulation mechanisms in glioma

Gliomas have long been recognized as immunosuppressive neoplasms that are characterized by the activation of various immune escape mechanisms. The coinhibitory characteristics of the PD-L1 molecule are attributed to the binding of this molecule to its receptor, PD-1, on tumor-specific T cells. This binding leads to apoptosis of the tumor-specific T cells and subsequently provides an immune escape for glioma cells that is similar to that of several types of extracranial tumors, including melanoma, lung cancer, gastric cancer, Ewing sarcoma, and head and neck cancer [21, 22]. The process of immunosuppression is correlated not only with the abnormal expression of PD-L1 on glioma cells but also with the microenvironment that tumor cells depend on. It has been reported that PD-L1 is expressed at a higher level in tumor-infiltrating macrophages in gliomas, which possibly leads to passive immunosuppressive effects due to T:T cell interactions [23]. Additionally, normal monocytes that are exposed to malignant glioma cells can significantly increase PD-L1 expression and assume a myeloid-derived suppressor cell (MDSC)-like phenotype. These MDSCs are characterized by the expression of PD-L1 and possess immunosuppressive activities, which result in the induction of apoptosis in activated T cells, and these cells have the ability to stimulate regulatory T cell proliferation [24]. More recently, PD-L1 expression in neuronal cells in the glioma microenvironment and post-transcriptional regulation by the endogenous production of interferon (IFN)-β have been reported. More importantly, PD-L1 expressed on neurons induces the caspase-dependent apoptosis of glioma cells, which results in longer survival times and suggests that the microenvironment can play a positive role in the inhibition of glioma growth [18].

**Fig. 1:**
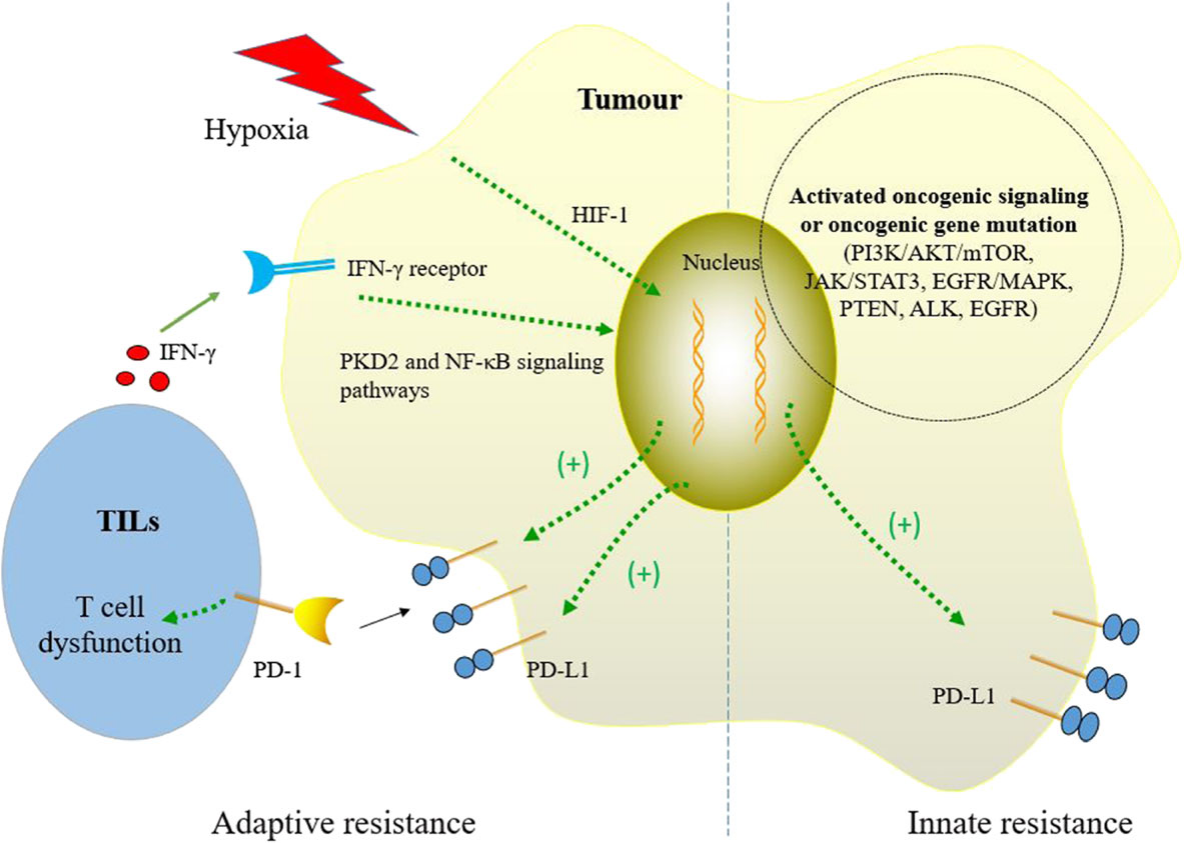
Adaptive resistance and innate resistance. (*Left*, adaptive resistance) Upon recognition of tumor antigens, TILs produce IFN-γ, which induces PD-L1 expression via nuclear NF-κB activation and the PKD2 signal pathway. In tumor hypoxia microenvironmental condition, HIF-1 regulates the expression of PD-L1 by binding directly to the hypoxia response element-4 in the PD-L1 proximal promoter. Upon binding to PD-1, PD-L1 delivers a suppressive signal to T cells, leading to T cell dysfunction. (*Right*, innate resistance) Tumor cell PD-L1 expression that might be related to oncogenic signaling pathways or oncogenic gene mutation as inherent in the tumor cell. Oncogenic signals (such as PI3K/Akt/mTOR, JAK/STAT 3, and EGFR/MAPK pathway) or oncogenic gene mutation (such as PTEN, ALK, and EGFR) upregulate PD-L1 expression on tumors as innate resistance. Abbreviations: *IFN-γ* interferon-γ, *TILs* tumor-infiltrating lymphocytes, *NF-κB* nuclear factor-kappaB, *PI3K* phosphatidylinositol 3-kinase, *HIF-1* hypoxia inducible factor-1, *JAK/STAT3* Janus kinase/signal transducer and activator of transcription 3, *EGFR/MAPK* epidermal growth factor receptor/mitogen-activated protein kinase, *ALK* anaplastic lymphoma kinase, *PKD2* polycystin 2, *PD-1* programmed death 1, *PD-L1* programmed cell death-ligand 1, *AKT* protein kinase B, *mTOR* mammalian target of rapamycin, *PTEN* phosphatase and tensin homolog

The upregulation of PD-L1 on tumor cells plays a significant role in the immune escape mediated by gliomas. The mechanisms of the upregulation of PD-L1 in gliomas are illustrated in Fig. 1. The upregulation of PD-L1-mediated evasion of tumor immunity has been termed “adaptive resistance,” stemming from the observations that extrinsic induction of PD-L1 is largely mediated by IFN-γ. IFN-γ is a proinflammatory cytokine mainly generated by T lymphocytes after antigen recognition and activation in adaptive immunity. Upon recognition of tumor antigens, T effector cells or tumor-infiltrating lymphocytes (TILs) produce IFN-γ, which drives PD-L1 expression in the tumor cells. Adaptive resistance is supported by flow cytometry-based observations that IFN-γ can induce high levels of cell surface PD-L1 expression in all 12 glioma cell lines [11]. In addition to the upregulation of PD-L1 in the protein level, IFN-γ could also increase the expression of PD-L1 mRNA [25]. IFN-γ induced PD-L1 transcription in lung carcinoma cells via binding on two interferon regulatory factor 1 sites (200 and 320 base pairs upstream of the transcriptional start site) in the promoter of PD-L1 [26]. Recent studies suggested that the activation of nuclear factor-kappaB is essential for IFN-γ-induced PD-L1 upregulation in human melanoma cells [27], and the PKD2 signal pathway is also involved in this upregulation on human oral squamous carcinoma [28]. Hypoxia is a well-recognized tumor microenvironmental condition. Hypoxia-inducible factor-1 plays a critical role in the regulation of cellular responses to hypoxia. It regulates the expression of PD-L1 by binding directly to the hypoxia response element-4 in the PD-L1 proximal promoter [29] (Fig. 1, left).

Constitutive oncogenic signals are shown to mediate intrinsic induction of PD-L1 as an “innate resistance” mechanism of immune evasion. This is evidenced by the small fraction of human cancers that lack TILs in the tumor microenvironment but still express high levels of PD-L1 [30, 31]. Parsa et al. [32, 33] measured the expression of PD-L1 in glioma cells and found that glioma cells with genetic deletions or mutations of the phosphatase and tensin homolog (PTEN) genes exhibit greater PD-L1 protein levels than cells with wild-type PTEN. Further research demonstrated that the PI(3)K-Akt-mTOR-S6K1 pathway increases the PD-L1 protein level, which results in gliomas that are inherently resistant to immunoreaction. So far, no general oncogenic signaling or oncogenic gene mutation is shown to mediate intrinsic induction of PD-L1. Depending on cell type, the expression of PD-L1 was found to correlate with various oncogenic signaling or oncogenic gene mutations, such as the Akt/mTOR, JAK/STAT 3, and EGFR/MAPK pathways [34–36] or PTEN, ALK, and EGFR mutations [37–39] (Fig. 1, right). MicroRNA (miRNA) is a small non-coding RNA molecule that functions in RNA silencing and post-transcriptional regulation of gene expression [40]. miR-34a and miR-200 have an inverse relationship with PD-L1 expression, which points to the role of epigenetic regulation in the regulation of PD-L1 in cancer cells [41, 42].

Recent findings have supported the notion that PD-L1 upregulation in tumor cells is related to both innate and adaptive resistance mechanisms. Han et al. [43] found that the expression levels of the PD-L1 transcript and protein are increased in both PTEN− and PTEN+ cell lines when the glioma cell lines are treated with IFN-γ. Additionally, IFN-γ induces significantly greater increases in the levels of PD-L1 protein and transcript in PTEN− tumor cells than in PTEN+ tumors. Coculture experiments have revealed that the activated oncogenic PI3K pathway participates in immune evasion through PD-L1 superinduction, which is mediated by IFN-γ in PTEN-deficient gliomas. In summary, these data indicate that complicated mechanisms of PD-L1 upregulation exist in gliomas owing to the unique tumor microenvironment and complex signaling pathways.

## Glioma treatment using a PD-1/PD-L1 blocking antibody

### Combination therapy strategy and preclinical research

The blockade of PD-1/PD-L1 can elicit effective anti-tumor T cell responses. In the past 5 years, the targeting of the PD-1/PD-L1 axis has been at the forefront of immunotherapy due to its remarkable clinical efficacy in melanoma and non-small cell lung cancer clinical trials [44, 45]. There is a growing interest in the development of combinatorial immunotherapy strategies for cancer treatment. An increasing number of preclinical studies in mouse models of GBM involving the orthotopic implantation of GL261 cells have demonstrated that combination treatment with PD-1 and a PD-L1 inhibitor can successfully treat the tumors.

Most of the preclinical research into gliomas involved targeting the PD-1/PD-L1 axis in addition to other immunosuppressive inhibitors. Huang et al. [46] reported the median survival of the mice that received the PD-1-inhibited natural killer (NK) cell treatment was prolonged to 44 longer days compared with 35 days in the NK cell treatment group and 29 days in the control group in an orthotopic glioma stem cell-like mouse model. The study indicated that the blockade of the PD-1/PD-L1 axis may promote the cotoxicity of NK cells against GSCs. Indoleamine 2,3-dioxygenase (IDO) is a tryptophan catabolic enzyme that is overexpressed in both antigen-presenting cells and tumor cells and enables tumor cells to escape from the immune response. The expression of IDO has been described in 96% of GBMs and is correlated with overall patient survival [47]. Wainwright et al. [48] administered 1-MT (an IDO inhibitor) alone or in combination with a cytotoxic T lymphocyte-associated antigen-4 (CTLA-4) mAb, a PD-L1 mAb, or both CTLA-4 and PD-L1 mAbs to mice with orthotopically implanted GL261 cells. The results demonstrated that only the group that was treated with the combination of all three, i.e., the PD-L1 mAb, the CTLA-4 mAb, and 1-MT, exhibited significantly better survival. Additionally, this group exhibited a significant immune response as demonstrated by the lowest level of immunosuppressive regulatory T cells and the highest levels of CD4+IFN-γ and CD8+IFN-γ.

Combination treatment with a PD-1/PD-L1 inhibitor and radiotherapy (RT) is an attractive option given the potential for increased tumor antigen release and presentation. RT counteracts the immunosuppressive tumor microenvironment by enhancing the presentation of normally suppressed tumor-associated antigens, increasing the expression of major histocompatibility complex class I and proinflammatory cytokines, promoting dendritic cell maturation and downregulating Fas ligand expression. RT can foster dendritic cell maturation and promote CD8+ T cell recruitment into tumors [49]. In preclinical trials, anti-PD-1/PD-L1 combined with RT improved local control and survival, and this finding provided an important developmental direction for combined treatment. Zeng et al. [50] tested the combination of anti-PD-1 immunotherapy and stereotactic radiosurgery (SRS) in an orthotopically implanted GL261 cell mouse model. With the RT plus anti-PD-1 treatment, the median survival was prolonged to 52 days (*P* < 0.001) compared with 27 days in the RT-alone group and 30 days in the anti-PD-1 monotherapy group, and 15 to 40% of the mice became long-term survivors (>90 days after implantation). On day 21 after implantation, infiltration by cytotoxic T cells had increased and regulatory T cell levels had decreased in the combined treatment group compared with the single-modality arms. T cell immunoglobulin mucin-3 (TIM-3) is another immune checkpoint molecule and acts as a negative regulator of the immune system [51]. Kim et al. [52] evaluated a combination of anti-TIM-3 antibody and anti-PD-1 immunotherapy and SRS in an orthotopic mouse GBM model. The triple-modality treatment (anti-PD-1 + SRS + anti-TIM-3) yielded a robust increase in the OS of 100% by day 146 (*P <* 0.05) compared with the other treatment arms. This triple-modality treatment increased the infiltrations of IFN-γ+ and tumor necrosis factor-α+ CD4 T cells as well as IFN-γ+ CD8 lymphocytes into the tumor. These findings indicate that anti-PD-1/PD-L1 pathway treatment combined with SRS may be a feasible treatment strategy in gliomas.

Bevacizumab is a humanized mAb that targets vascular endothelial growth factor and became the third drug approved by the FDA for use in recurrent GBM in 2009 [53]. However, the addition of bevacizumab to standard therapy in newly diagnosed glioblastoma patients has demonstrated no improvement in OS [54]. The clinical responses of anti-VEGF treatment are transient; clinical relapse usually occurs within months after an initial response. By contrast, cancer immunotherapies can elicit durable and striking clinical activities [55]. Thus, it is reasonable to assume that the combination of bevacizumab with immunotherapy is a favorable approach. Indeed, combined therapy using PD-1/PD-L1 axis blockade and anti-VEGF treatment has demonstrated encouraging antitumor activity and tolerable adverse events in some animal models and in clinical studies [56, 57]. There are also some crucial considerations to account for in the development of targeted therapy and immunotherapy combinations, which include the optimization of dosing regimens and the minimization of treatment-related toxicities.

### Clinical studies of PD-1/PD-L1 targeting in glioma

#### Clinical trials

Clinical trials have been initiated to determine the potential of PD-1/PD-L1 checkpoint inhibitors as monotherapies and combination therapies for glioblastomas (Table 2).

**Table 2:** Summary of current PD-1 and PD-L1 blockade agents in clinical trials

Target	Clinical trial identifier	Blockade agent	Phase	Patient population	Design	Study start date	Current stage
PD-1	NCT02550249	Nivolumab	II	Primary and recurrent GBM	Preoperative neoadjuvant	June 2015	Recruiting participants
	NCT02423343	Nivolumab	I/II	Recurrent or refractory NSCLC HCC GBM	Combination with galunisertib	October 2015	Recruiting participants
	NCT02017717 (CheckMate 143)	Nivolumab	III	Recurrent GBM	Alone or in combination with the IPI Compared to bevacizumab	January 2014	Ongoing
	NCT02311920	Nivolumab	I	Primary GBM	IPI and/or NIVO in combination with temozolomide	April 2015	Recruiting participants
	NCT02337491	Pembrolizumab	II	Recurrent GBM	With or without bevacizumab	February 2015	Ongoing
	NCT02311582	Pembrolizumab	I/II	Recurrent GBM	Combination with MRI-guided laser ablation	August 2015	Recruiting participants
	NCT01952769	Pidilizumab	I/II	DIPG and recurrent GBM	Alone	February 2014	Recruiting participants
PD-L1	NCT02336165	MEDI4736	II	Primary and recurrent GBM	Combination with radiotherapy and bevacizumab	February 2015	Recruiting participants
	NCT01375842	MPDL3280A	I	Solid tumors (include GBM)	Alone	June 2011	Recruiting participants

This information of clinical trials came from the web site of clinicaltrials.gov (The last search was conducted on October 15, 2016).

*Abbreviations*: *GBM* glioblastoma, *NSCLC* non-small cell lung cancer, *HCC* hepatocellular carcinoma, *NIVO* nivolumab, *IPI* ipilimumab, *DIPG* diffuse intrinsic pontine glioma

NIVO is a fully human IgG4 subtype antibody that targets human PD-1 with a stabilizing hinge region mutation that is resistant to the exchange of IgG4 molecules. The FDA has approved NIVO for unresectable or metastatic melanomas and NSCLCs. NIVO is currently being investigated as a monotherapy for GBM in a phase II trial (NCT02550249). Ongoing trials of NIVO in combination with galunisertib (a kinase inhibitor of TGFβRI) are being conducted in patients with GBM (NCT02423343). Ipilimumab (IPI) is a fully humanized mAb against CTLA-4 and was approved by the FDA in 2011 for advanced melanoma [58–60]. Based on the safety and efficacy observed in melanoma [61], the combination of IPI and NIVO was tested in recurrent GBM. This was a phase III randomized trial (CheckMate 143, NCT02017717) that enrolled patients with GBM on December 17, 2013, and the study began on January 6, 2014. The updated results presented in 2016 at the American Society of Clinical Oncology (ASCO) Annual Meeting demonstrated encouraging efficacy outcomes in the completed phase I cohorts 1 and 1b. Among 40 patients with a first recurrence of GBM following radiation and temozolomide, 20 patients (cohort 1) were randomized 1:1 to NIVO 3 mg/kg (N3) every 2 weeks (Q2W) or NIVO 1 mg/kg + IPI 3 mg/kg every 3 weeks (Q3W; N1 + I3) for four doses followed by N3 Q2W. Twenty patients in cohort 1b received NIVO 3 mg/kg + IPI 1 mg/kg Q3W (N3 + I1) for four doses followed by N3 Q2W. Stable disease or better was achieved in 6/10, 4/10, and 9/20 patients who were treated with N3, N1 + I3, and N3 + I1, respectively. The OSs at 12 months were 40% (95% CI 12–67), 30% (95% CI 7–58), and 25% (95% CI 8–48) in the N3, N1 + I3, and N3 + I1 groups, respectively [62]. Furthermore, NIVO in combination with IPI and chemotherapy will be tested in patients with GBM. There is an ongoing phase I safety study that was planned to investigate IPI and NIVO in combination with temozolomide for recurrent GBM (NCT02311920).

Pembrolizumab is a humanized monoclonal IgG4 anti-PD-1 antibody consisting of a high-affinity mouse anti-PD-1-derived variable region grafted onto a human IgG4 immunoglobulin molecule with an engineered Fc region for stabilization. Pembrolizumab was approved in 2014 by the FDA for the treatment of patients with IPI-treated advanced melanoma [63]. In a recent phase II clinical trial, patients with untreated brain metastases from melanomas or NSCLCs were treated with 10 mg/kg pembrolizumab every 2 weeks until progression. Responses of the CNS lesions were achieved in four (22%; 95% CI 7–48) of 18 patients with melanoma and in six (33%; 95% CI 14–59) of 18 patients with NSCLC [64]. Pembrolizumab is currently being tested in combination with bevacizumab (NCT02337491) and with MRI-guided laser ablation (NCT02311582) in patients with recurrent GBM. Additionally, the anti-PD-1 antibody pidilizumab is a humanized mAb that modulates the immune response, and evaluation in patients with metastatic melanoma revealed an OS at 12 months of 64.5% [65]. A randomized phase I/II study has been planned to test the effect of pidilizumab against diffuse intrinsic pontine glioma and recurrent GBM (NCT01952769).

In addition to the PD-1 inhibitors discussed above, there are two anti-PD-L1 agents that are currently being evaluated in clinical trials for gliomas. The human anti-PD-L1 antibody MEDI4736 has demonstrated a durable response in patients with melanoma and NSCLC [66], and MEDI4736 is now being tested in combination with radiotherapy and bevacizumab in the treatment of GBM (NCT02336165). MPDL3280A, which is also a human anti-PD-L1 monoclonal antibody, has received a breakthrough therapy designation from the FDA for PD-L1-positive urothelial bladder cancer and NSCLC. A current phase I study is investigating the safety and pharmacokinetics of MPDL3280A as administered as a single agent to patients with solid tumors, including GBM (NCT01375842). The relative paucity of clinical trials of anti-PD-L1 inhibitors may be because PD-L1 is located inside tumor cells. PD-L1 inhibitors would have to penetrate both the blood-brain barrier and the blood-tumor barrier to be effective [67]. Moreover, because the expressions of PD-L2 and possibly other tumor-associated molecules may play roles in tolerating PD-1-expressing lymphocytes, the magnitude of the anti-tumor immune response could also be blunted [68].

#### Adverse events

The aim of antibodies that target either PD-1 or PD-L1 is to block the PD-1/PD-L1 pathway with the goal of adjusting and normalizing immunity to a desirable level while not enhancing immunity in general [69]. The achievement of this aim may provide an explanation as to why PD-1 and PD-L1 inhibitors have resulted in dramatic clinical efficacies with reduced toxicities. To date, data regarding the adverse events associated with these glioma treatments are still limited. There is only one report of a clinical trial (CheckMate 143), which was presented at the 2015 and 2016 ASCO Annual Meetings [62, 70]. The preliminary results regarding drug-related adverse events from a phase I safety cohort 1 trial were reported at the 2015 ASCO Annual Meeting [70]. The events associated with N3 were all grade 1 or 2 and included fatigue (*n* = 3) and nausea (*n* = 3). For the N1 + I3 group, the adverse events included fatigue (*n* = 8), diarrhea (*n* = 7), increases in glutamic oxalacetic aminopherase and lipase (*n* = 5 each), increases in vomiting and alanine aminotransferase (*n* = 4 each), and amylase increase, headache, hyperthyroidism, nausea, and maculo-papular rash (*n* = 3 each). Among these N1 + I3 patients, 8/10 developed grade 3–4 adverse events. Treatment discontinuation due to drug-related adverse events, which included colitis, cholecystitis, diabetic ketoacidosis, confusion, and increased lipase, occurred in only 5 patients with N1 + I3. Updated results regarding adverse events were presented at the 2016 ASCO Annual Meeting [62]. In the N3, N1 + I3, and N3 + I1 groups, any-grade treatment-related adverse events were reported in 9/10, 10/10, and 20/20 patients, respectively, and the corresponding proportions for grade 3–4 adverse events were 0/10, 9/10, and 5/20. In the N3, N1 + I3, and N3 + I1 groups, any-grade treatment-related serious adverse events were observed in 2/10, 7/10, and 5/25 patients, respectively, and the corresponding proportions for grade 3–4 adverse events were 0/10, 7/10, and 2/20. Treatment discontinuation due to treatment-related adverse events was required in none of the N3 patients, 3 of the N1 + I3 patients, and 1 of the N3 + I1 patients. Encouragingly, no treatment-related deaths have occurred in this cohort. Adverse events associated with PD-1 inhibition in patients with brain metastases in a phase II trial have been reported in detail, and these results could provide important information [64]. These results demonstrated that pembrolizumab was well tolerated in 36 patients with brain metastases (18 with melanoma and 18 with NSCLC). In the melanoma cohort, only a single patient developed severe adverse events (grade 3), and the grade 1–2 adverse events were fatigue (*n* = 8), anorexia (*n* = 1), dermatological issues (*n* = 6), arthralgias (*n* = 2), and endocrine problems (*n* = 1). In the NSCLC cohort, the serious adverse events included (one patient each) acute kidney injury (grade 2), pneumonitis (grade 3), colitis (grade 3), hyperkalemia (grade 4), and fatigue (grade 3). The grade 1–2 adverse events included colitis or diarrhea (*n* = 3), acute kidney injury (*n* = 1), fatigue (*n* = 5), anorexia (*n* = 3), dermatological problems (*n* = 4), arthralgias (*n* = 1), endocrine problems (*n* = 5), and hematologic effects (*n* = 2). Neurological adverse events were also reported in the clinical trial, and to the best of our knowledge, data regarding PD-1 and PD-L1 inhibitors remain scarce [64]. The neurological adverse events were grades 1–2, and none led to treatment discontinuation. Eight of 18 patients in the melanoma cohort developed neurological adverse events that included grade 3 cognitive dysfunction (*n* = 1), grade 1–2 seizures (*n* = 3), headache (*n* = 3), and dizziness (*n* = 1); in addition, 2 of these patients developed neurologic symptoms due to peri-lesional edema (1 developed grade 3 cognitive dysfunction, and 1 developed grade 2 seizures). The neurological adverse events in the NSCLC cohort were grades 1–2 and included cognitive dysfunction (*n* = 1), headache (*n* = 4), dizziness (*n* = 2), and stroke (*n* = 1). There were no treatment-related deaths or autoimmune events, which indicated the safety of the blocking PD-1 or PD-L1 antibodies in patients with CNS tumors.

#### Current challenges

There are some challenges to the clinical application of targeting the PD-1/PD-L1 axis as a therapeutic modality in patients with glioma. First and the most important, biomarkers that identify patients who are likely to respond to PD-1 or PD-L1 inhibition have not been defined. Although PD-L1 immunohistochemistry has been approved by the FDA as the only predictive companion diagnostic test for the use of pembrolizumab in NSCLC patients, improved survival outcomes have been observed in many PD-L1-negative patients [71]. Additionally, standardized methods for the detection of PD-L1 and a scoring cut-off for determining PD-L1 positivity in glioma cells are lacking. Second, the criteria for the assessment of the responses of solid tumors to conventional therapy are the Response Evaluation Criteria, which may not be well suited to immunotherapy. Immunotherapy Response Assessment in Neuro-Oncology criteria are currently being drafted by a multinational panel to standardize the response assessment criteria for patients with neurooncological malignancies and to prevent premature termination of immunotherapies, including anti-PD-1/PD-L1 therapies, due to pseudoprogression [72]. Third, both preclinical and clinical studies of anti-PD-1/PD-L1 in gliomas are still limited. Last, research efforts have focused on anti-PD-1/PD-L1 therapies combined with other treatment modalities, including chemotherapy, molecular targeted agents, and RT, in gliomas. The optimal sequence of the combination and the suitable dose and fractionation of RT should be confirmed. Future research should aim to develop new effective agents or optimal combined treatment strategies that elicit the lowest toxicities to improve the outcomes of glioma.

## Conclusions

PD-L1 is expressed in glioma cells, correlates with tumor grade, and contributes to immunoresistance. The PD-1/PD-L1 pathway plays an important role in glioma biology. The use of anti-PD-1/PD-L1 antibodies in immunotherapeutic strategies for gliomas is attracting increasing attention. With the goal of improving the efficacy of anti-PD-1/PD-L1 immunotherapy, many studies have focused on combination treatment, including the targeting of multiple immune inhibitors, RT, ablation, chemotherapy, and other molecular targeting therapies. Because there is a need for better and safer treatment strategies for gliomas, the initiation of a greater number of successive clinical trials associated with immune checkpoint blockade along with further exploration into the mechanism of tumor immunity would be warranted.

## Abbreviations

ALK: Anaplastic lymphoma kinase
CNS: Central nervous system
CTLA: Cytotoxic T lymphocyte-associated antigen
EGFR/MAPK: Epidermal growth factor receptor/mitogen-activated protein kinase
FDA: Food and Drug Administration
GBM: Glioblastoma
GSCs: Glioma stem-like cells
HGG: High-grade glioma
IDO: Indoleamine 2,3-dioxygenase
IFC: Immunofluorescence histochemistry
IFN-γ: Interferon-γ
IHC: Immunohistochemistry
IPI: Ipilimumab
JAK/STAT 3: Janus kinase/signal transducer and activator of transcription 3
LGG: Low-grade glioma
mAb: Monoclonal antibody
MDSCs: Myeloid-derived suppressor cells
MRI: Magnetic resonance imaging
NIVO: Nivolumab
NK: Natural killer
NM: Not mentioned
NSCLC: Non-small cell lung cancer
OS: Overall survival
PD-1: Programmed death-1
PD-L1: PD-ligand 1
PE: Paraffin-embedded specimens
PTEN: Phosphatase and tensin homolog
RT: Radiotherapy
SRS: Stereotactic radiosurgery
TCGA: The Cancer Genome Atlas
TILs: Tumor-infiltrating lymphocytes
TIM-3: T cell immunoglobulin mucin-3
WB: Western blot
WHO: World Health Organization
